# The impact of digital presence and use of information technology on business performance of veterinary practices: a case study of Bosnia and Herzegovina

**DOI:** 10.3389/fvets.2023.1208654

**Published:** 2023-09-01

**Authors:** Nihad Fejzić, Abdullah Muftić, Sabina Šerić-Haračić, Emina Muftić

**Affiliations:** ^1^Department of Pathobiology and Epidemiology, Veterinary Faculty, University of Sarajevo, Sarajevo, Bosnia and Herzegovina; ^2^Department of Bromatology and Nutrition, Faculty of Pharmacy, University of Sarajevo, Sarajevo, Bosnia and Herzegovina

**Keywords:** digital presence, business performance, management, marketing, information technologies, veterinary practice

## Abstract

**Introduction:**

The veterinary profession is facing various challenges in the 21st century, such as livestock production intensification, shifting distribution of infectious diseases, growing focus on food safety alongside growing demand for companion animals’ veterinary services. Information technologies and digitalization trends had driven changes in many business sectors, including providing veterinary services thus opening new avenues to overcome the existing challenges this profession is facing.

**Methods:**

A study was conducted among 244 veterinary practices in Bosnia and Herzegovina. The gathered information related to subjects involving digital presence, the utilization of information technologies, and the business performance. To obtain this information, a personalized questionnaire was utilized as the means for collecting data.

**Results:**

The study revealed that only 10.2% had a business-associated website, and 54.9% were present on at least one social media platform. The study suggests that a positive impact on annual profit can be achieved through the implementation of effective digital marketing strategies such as web presence, search engine optimization, Google business account existence, website Google advertisement, continuous administration of social media, and social media advertisement. The statistical analysis indicates that Google advertisements, website search engine optimization, and social media advertisements greatly affect annual profit.

**Discussion:**

Improving digital presence of veterinary businesses with professionally managed websites, use of social media platforms, investment in online marketing strategies, and adopting telehealth services and online access to patient records positively affects business performance and better fits the growing needs of clients and society.

## 1. Introduction

The 21st century has brought a number of challenges to the veterinary profession. The increasing demand for animal protein has significantly impacted livestock production systems as the human population grows. The intensification of production and increased trade of animals (terrestrial, aquatic, and wildlife) and their products have led to increased risk and burden of transboundary, emerging and re-emerging infectious diseases, including zoonotic ones (1). The requirements to improve farms’ biosecurity, disease surveillance, reporting, and development of new preventive approaches become important more than ever. From a consumer perspective, food safety is one of the major concerns. On the other side, there is a continuing and growing demand to satisfy needs in sophisticated health care and clinical practice for companion animals, with pets booming as a trend in our era (2, 3). This shift in focus has also led to an increase in the number of veterinary clinics and hospitals that focus exclusively on pet care. The veterinary profession is increasingly being recognized as a vital part of the healthcare system for companion animals, food producing animals and public health, is in high demand in the global work market. Today there is a significant shortage of veterinarians across all sectors of professional activities in both food production and companion animals (3, 4).

The adoption of information technologies and digital transformation of business are crucial in today’s world and will continue to be, as they enable innovation, efficiency, and competitiveness across all industries and sectors (5). Veterinary medicine and the profession are not isolated from acknowledging the value and potential of those technologies, notably in addressing the numerous challenges, including the shortage of qualified professionals and the growing demand for diversification and highly specialized veterinary services. Yet, there is a lack of studies analyzing and proposing how to soundly and most effectively integrate and adopt new technologies in veterinary practices, in order to maximize their potential and addressgrowing needs of veterinary services consumers. The objectives of this study were to:

- Evaluate the current status of digital presence and related use of information technology in veterinary practices in Bosnia and Herzegovina.
- Investigate the impact of digital presence and information technology on the business performance of veterinary practices, and
- Initiate the development of guidelines and activities for the effective adoption and implementation of certain digital technologies in order to improve the delivery of veterinary tasks together with enhancing the existing knowledge and skills of veterinarians.

## 2. Materials and methods

In the study we investigated all registered veterinary practices in the country using data base of the central competent authority (Veterinary Office of Bosnia and Herzegovina). Only those being business active in 2021 were considered for analysis, leading to the study sample of 244 veterinary practices. Data on use of information technologies and digital presence of veterinary business were collected using check list made for the purpose of this study (Table 1). The check list included 39 individual data entry for each practice, categorized into five categories, completed by investigators using different online sources. Selection of data sources was made based on the criteria of comprehensiveness, relevance and accessibility of data required for the study. Digital sources, the categories, and description of data collected were as follows:

a) Website performance: this category includes the existence of a website, the type of programming language used to develop the website (such as JavaScript, Angular, React, etc.), the continuous administration of the website, the availability of telehealth services, online appointment booking, online access to patient records, the existence of an online shop, and the availability of a customer review panel; sources used: businesses websites, www.google.com, www.SEMrush.com. Directly accessing and analyzing the websites of the veterinary practices themselves allowed for a firsthand evaluation of their online presence, features, and functionalities. This source provides authentic and up-to-date information about the existence of a website, the type of programming language used, and the presence of online shop and customer review panels. Google and SEMrush are widely recognized tools are industry-standard platforms for digital marketing and SEO analysis. SEMrush provides valuable insights into website traffic, keywords, and SEO performance, while Google’s tools enable the assessment of telehealth services, online appointment booking, and access to patient records. The use of these tools ensures the accuracy and reliability of the data related to website performance.
b) Social media performance: this category covers the existence of profiles on platforms such as Facebook, Instagram, LinkedIn, and TikTok, the professional management of these accounts, the continuous administration of the pages, communication with the business and the response rate, the availability of appointment booking, posts promoting patient treatments and employee education, and posts related to other public engagement actions; sources used: www.Facebook.com, www.Instagram.com, www.TikTok.com, and www.LinkedIn.com. These social media platforms are among the most popular and widely used in the world. By evaluating the existence and management of veterinary practice profiles on these platforms, the study gained valuable information about their social media presence and engagement with the public. The communication with the business and response rate on these platforms was also assessed, providing insights into their responsiveness to customer inquiries.
c) Digital marketing performance: this category includes website advertisement, social media advertisement, search engine optimization (SEO), the website’s ranking compared to the world’s average in the category “health” in terms of SEO, the percentage of organic traffic, and the percentage of advertised traffic; sources used: https://business.facebook.com/, and www.SEMrush.com. Facebook business is a dedicated platform for businesses to manage and analyze their advertisements on Facebook and Instagram. By using this platform, the study obtained data on website advertisement and social media advertisement strategies adopted by veterinary practices. SEMrush, as mentioned earlier, is a powerful tool for analyzing online marketing performance, including SEO, organic traffic, and advertised traffic, providing data to assess digital marketing effectiveness.
d) Mobile application performance: this category evaluates the existence of a mobile application, its rating, and whether the application is primarily focused on shopping. Sources used: www.apple.com/app-store, www.play.google.com/store/apps. As the leading app distribution platforms for iOS and Android devices, respectively, the Apple App Store and Google Play Store were used to gather data on the existence of mobile applications for veterinary practices. Additionally, these platforms provided information on the rating of the applications, offering insights into their popularity and user satisfaction.
e) Business performance: this category covers financial performance, such as the annual revenue of the business, the annual profit, the existence of financial blockades, the difference in capital goods compared to the previous year, the existence of a Google business account, and the business’s rating on Google. All of the reported financial reports use the convertible mark (BAM), the national currency Bosnia and Herzegovina. Sources used: www.companywall.ba, www.google.com/business. Company Wall is a reputable online platform that provides information about businesses, including their financial performance and ratings. The study used this platform to access data on the annual revenue and profit of veterinary practices in Bosnia and Herzegovina. Google Business, on the other hand, offers insights into business ratings and the presence of a Google business account, which were relevant metrics for assessing the overall performance of veterinary businesses.

**Table 1:** Questionnaire for data collection on digital presence, information technologies utilization and business performance from veterinary practices in Bosnia and Herzegovina.

Variable type	Variable name	Description
—	Data ID	A unique identification code (ID) for the survey (for each business)
—	Name	Name of the business
—	Entity	Entity in Bosnia and Herzegovina: Republic of Srpska (RS), Federation of BiH (FBiH), Brcko District (BD)
—	County	County in Bosnia and Herzegovina: name of the county
Dichotomous	Website	Does the website of the business exist? 1 (Yes), 0 (No)
Dichotomous	Website creation	Is website made utilizing native JavaScript, React, Angular or similar programming languages? Positive answer indicates that website is professionally made. Using WordPress and other website building tools, whilst using JavaScript as well, have higher latency rates, lower search engine optimization and could be done by the amateur. Checking of programming language utilization was performed by analysis of the metadata of the webpage 1 (Yes), 0 (No)
Dichotomous	Website administration	Does website receive continuous administration (last post <30 days ago)? 1 (Yes), 0 (No)
Dichotomous	Services observable	Are the services and corresponding prices by the business observable on the website? 1 (Yes), 0 (No)
Dichotomous	Telehealth	Is telehealth available? 1 (Yes), 0 (No)
Dichotomous	Email	Does email exist as the mean of communication on website? 1 (Yes), 0 (No)
Quantitative (discrete)	Email response time	How fast does the business respond to an email sent towards them? Number of days
Dichotomous	Contact form	Does the webpage contact form exist as the mean of communication on website? 1 (Yes), 0 (No)
Quantitative (discrete)	Contact form response time	How fast does the business respond to the filled contact form on the website? Number of days
Dichotomous	Patient records online	Can you access patient records online? 1 (Yes), 0 (No)
Dichotomous	Customer review panel	Does the customer review panel exist? 1 (Yes), 0 (No)
Dichotomous	Online shop	Does online shop exist? 1 (Yes), 0 (No)
Dichotomous	Facebook	Does the business have Facebook page? 1 (Yes), 0 (No)
Dichotomous	Instagram	Does the business have Instagram page? 1 (Yes), 0 (No)
Dichotomous	TikTok	Does the business have TikTok page? 1 (Yes), 0 (No)
Dichotomous	LinkedIn	Does the business have LinkedIn page? 1 (Yes), 0 (No)
Dichotomous	Social media management	Are the social media accounts managed professionally? 1 (Yes), 0 (No)
Dichotomous	Social media administration	Do social media pages receive continuous administration (last post <15 days ago)? 1 (Yes), 0 (No)
Dichotomous	Chatting with business	Is it available to chat with the business via social media? 1 (Yes), 0 (No)
Quantitative (discrete)	Chatting response time	How long does it take for business administrator to answer a question directed via the chat? Number of hours
Dichotomous	Social media appointment booking	Is it possible to book an appointment via social media? 1 (Yes), 0 (No)
Dichotomous	Social media advertising	Does the business use social media to advertise? Instagram and Facebook advertising surveyed through the Facebook business account 1 (Yes), 0 (No)
Dichotomous	Social media self-promotion	Are social media used to promote successful treatments of the patients and/or education of the employees? 1 (Yes), 0 (No)
Dichotomous	Social media public engagement	Are any type of public engagement actions by the business present at the social media accounts? 1 (Yes), 0 (No)
Dichotomous	Business website advertising	Does business advertise the website? Advertising is surveyed via specialized site www.semrush.com 1 (Yes), 0 (No)
Dichotomous	Search engine optimization	Is website SEO (search engine optimized)? SEO is surveyed via specialized site www.semrush.com 1 (Yes), 0 (No)
Dichotomous	Search engine ranking	Does the website rank better in terms of SEO than average website in the category “health” (79%)? SEO ranking is surveyed via specialized site www.semrush.com 1 (Yes), 0 (No)
Dichotomous	Organic traffic	Percentage of organic traffic? Organic traffic is the traffic that comes from accessing the site from visitor searches on Google, Yahoo, Bing, or other search engines. Traffic is surveyed via specialized site www.semrush.com 1 (Yes), 0 (No)
Dichotomous	Advertising traffic	Percentage of advertising traffic? Advertising traffic is the traffic that reaches a site via a display ad through an ad network. Traffic is surveyed via specialized site www.semrush.com 1 (Yes), 0 (No)
Dichotomous	Mobile application	Is there a mobile application developed and deployed by the business? Mobile application presence is established through search at Google Play Store and iPhone App store 1 (Yes), 0 (No)
Dichotomous	Mobile application shop	Is the mobile app made with focus on presenting current retail offers of the business to the users while promoting sales via shop? 1 (Yes), 0 (No)
Ordinal	Mobile application rating	What is the rating of the mobile app on the digital distribution service? It corresponds with the review scores by the users of the mobile app. Rank: 1–5 (decimal), if below 0.5 round down, otherwise round up
Dichotomous	Financial account blocked	Does the business have blocked financial account? Blocked financial account is repercussion of insolvency or liquidation of the business. Financial data is surveyed via specialized site www.companywall.ba 1 (Yes), 0 (No)
Quantitative (discrete)	Annual revenue of the business	What is the annual revenue of the business for the last fiscal year (2021)? Amount of Bosnian mark (BAM)
Quantitative (discrete)	Annual profit of the business	What is the annual profit of the business for the last fiscal year (2021)? Amount of Bosnian mark (BAM)
Quantitative (discrete)	Capital goods	Difference between this year’s and last year’s business capital goods? Together with the annual profit gives strong estimation of the business success amount of Bosnian mark (BAM)
Quantitative (discrete)	Number of registered workers	How many workers are registered in the last fiscal year (2021)? Number of people
Dichotomous	Google business account	Does the business have Google business account created? Google business account allows owners to add their site to the google maps platform, as well as to participate in Google advertising? 1 (Yes), 0 (No)
Ordinal	Google rating	What is the rating of the business on Google? It corresponds with the review scores by the visitors. Rank: 1–5 (decimal), if below 0.5 round down, otherwise round up

Stratification of data collected was further utilized in the statistical analysis and comparisons. Statistical analysis was performed by *t*-tests and Hedges’ *g* tests utilizing R Project for Statistical Computing. *t*-tests were used to compare the means of the different digital technologies on business performance, and Hedges’ *g* tests were used to evaluate the size of the effect established by *t*-test. Thresholds for Hedges’ *g* test were as follows: <0.6—small effect; ≤0.8—medium effect; ≥0.81—large effect. Annual profit was used as a dependent variable by which effects of all of the other independent variables were evaluated.

### 2.1. Ethical considerations

It is important to note that there are some limitations in this study, mainly regarding the reported revenue and profit by the business, which could differ from real data. Additionally, ethical considerations were taken into account during the research to ensure the protection of the participant’s private information.

## 3. Results

### 3.1. Digital presence and information technology adoption

**Figure 1:**
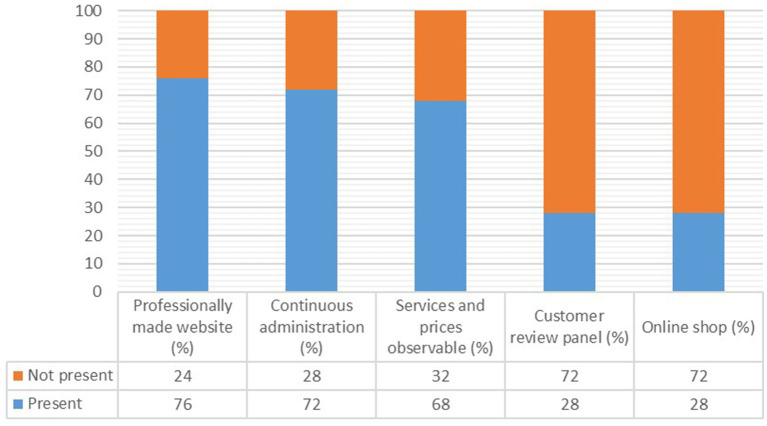
Proportional representation of website features on veterinary practices websites in Bosnia and Herzegovina.

A total of 244 veterinary practices were surveyed to assess their utilization of online platforms, websites, social media, and digital marketing strategies. We found that a relatively small proportion of veterinary practices in Bosnia and Herzegovina had established a business-associated website, with only 10.2% of the surveyed practices having an online presence. Among those with a website, a mere 7.8% had their websites professionally created and managed, indicating a lack of emphasis on digital branding and presentation. The majority of the websites (72%) served as an informational and booking front for the businesses, providing essential information about the services offered but lacking in interactive features. Only 28% of the websites had an online shop and customer review panel, indicating a limited focus on e-commerce and customer engagement (Figure 1).

**Figure 2:**
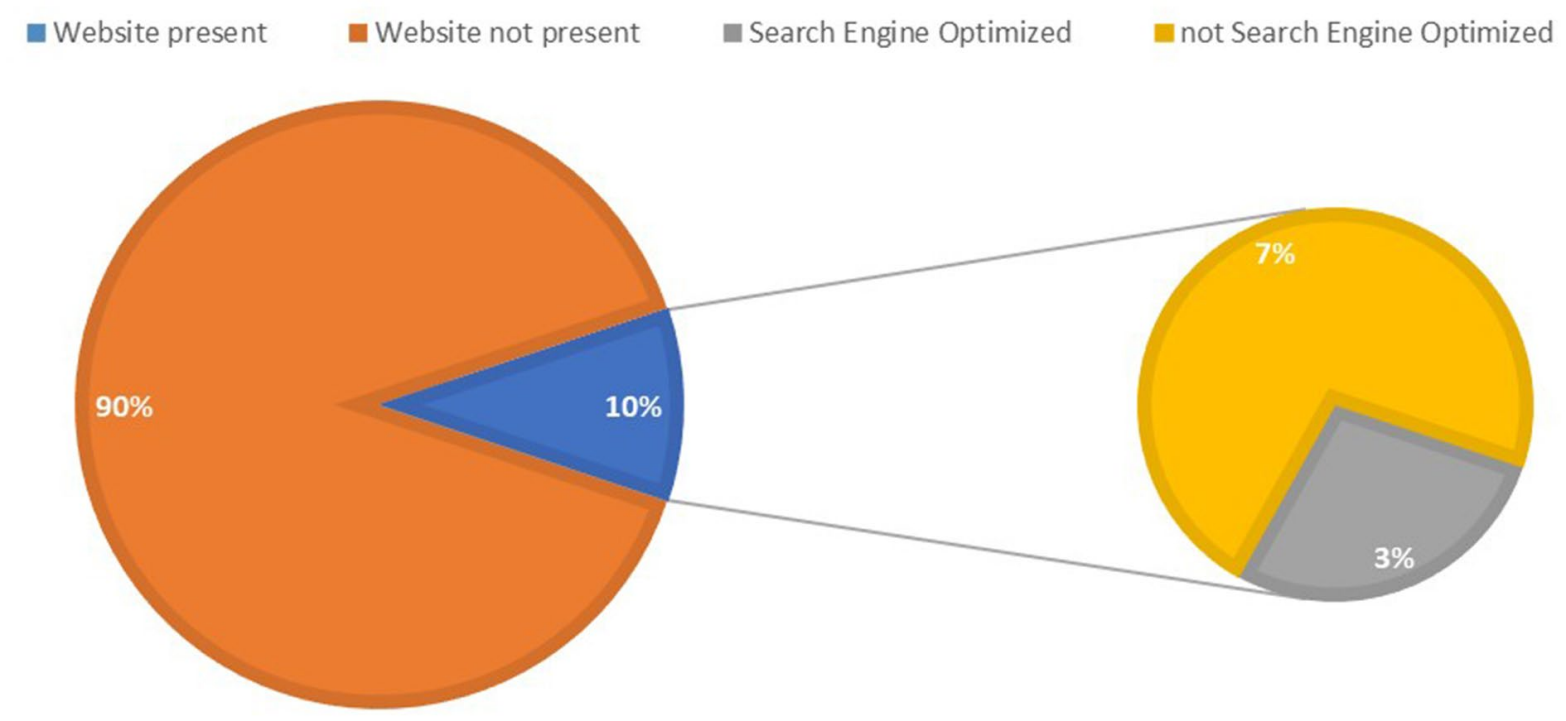
Existence of businesses’ websites of veterinary practices in Bosnia and Herzegovina and websites’ search engine optimization.

Furthermore, it was observed that only a minority of the websites had been optimized for search engines, suggesting a lack of awareness of the potential benefits of SEO in enhancing online visibility and attracting potential clients (Figure 2). Additionally, just 24% of the surveyed websites ranked higher than the global average for websites in the “health” category.

Regarding communication methods employed by website-owning veterinary practices, email correspondence and booking were the most prevalent, with 76% of practices using this approach. This was followed by the use of contact forms (72%) for communication. However, the average response times for these methods were 3.11 and 2.56 days, respectively, indicating potential areas for improvement in responsiveness and customer service. Notably, none of the surveyed veterinary practices had adopted telehealth services or provided online access to patient records.

**Figure 3:**
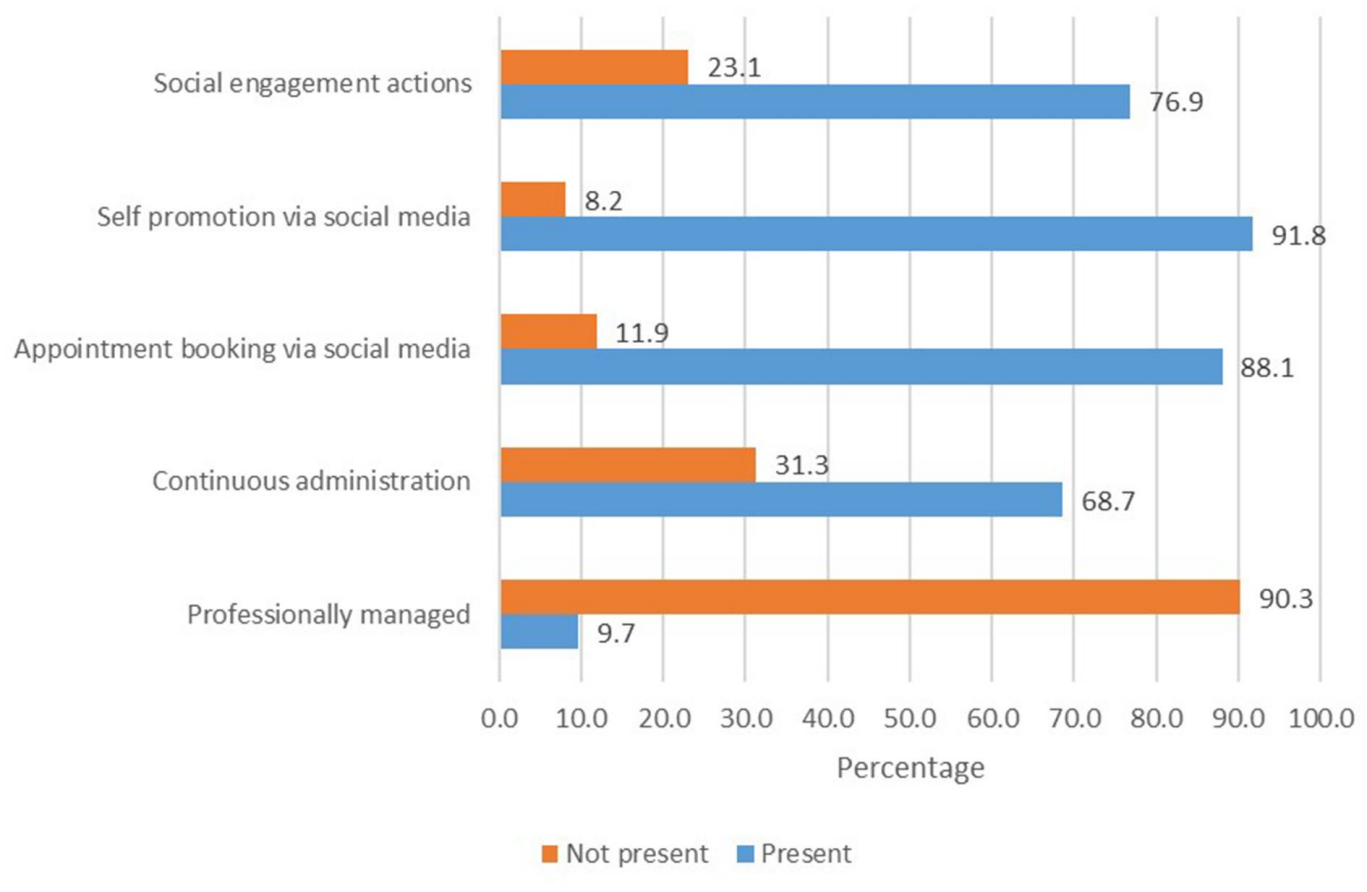
Proportional representation of social media practices among veterinary businesses in Bosnia and Herzegovina with social media presence.

Social media platforms also played a role in the digital presence of veterinary practices. Approximately 54.9% of the surveyed businesses maintained a presence on one or more social media platforms. Among these, Facebook emerged as the most prevalent platform, being utilized by all businesses with a social media presence. A smaller proportion of businesses had accounts on Instagram (11.9%) and LinkedIn (0.8%). Interestingly, none of the veterinary practices had a presence on TikTok, suggesting a lack of engagement with newer and potentially impactful social media platforms. The level of social media activity exhibited significant variation among the veterinary businesses that maintained a presence on these platforms (Figure 3). While some practices actively engaged with their audience through regular posts and updates, others seemed to underutilize the potential of social media as a communication and marketing tool.

**Figure 4:**
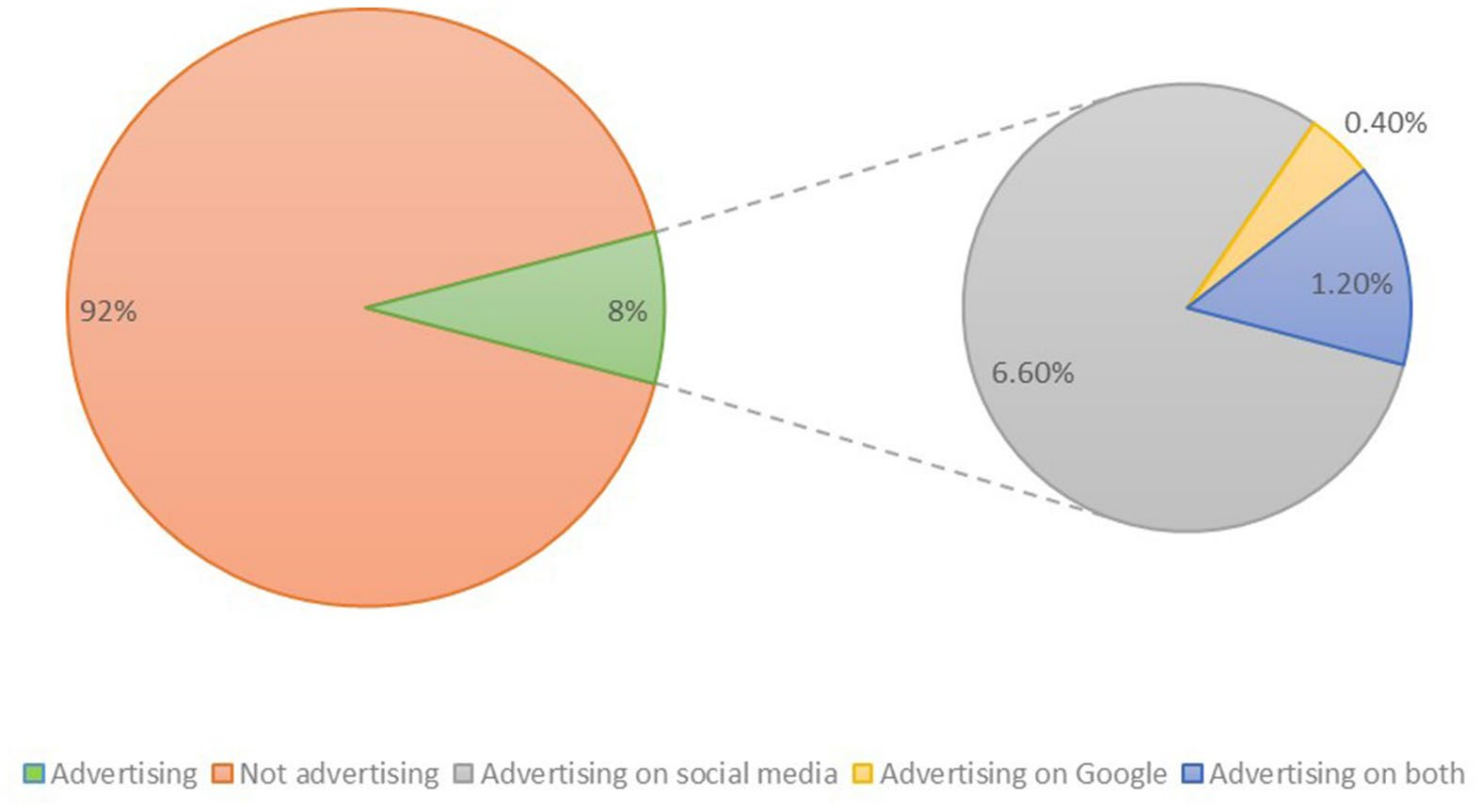
Proportional representation of veterinary practices in Bosnia and Herzegovina engaging in online advertising and their preferred online advertising platforms.

Digital marketing practices among veterinary businesses in Bosnia and Herzegovina were found to be relatively limited. Only 8.2% of the surveyed practices advertised via the internet, indicating a low adoption of digital marketing strategies. Among those practices that did advertise, social media advertising was the more popular choice compared to website advertising (Figure 4). Finally, the study assessed the presence of mobile applications among the surveyed veterinary businesses. None of the practices had deployed a mobile application on either the Apple App Store or Google Play Store. In summary, our study reveals that veterinary practices in Bosnia and Herzegovina have only partially embraced digital presence and information technology adoption. In summary, a noticeable deficiency was noted in almost all spheres of online presence, most noticeably: website development, SEO, telehealth services, and mobile application advancement.

### 3.2. Business performance

**Figure 5:**
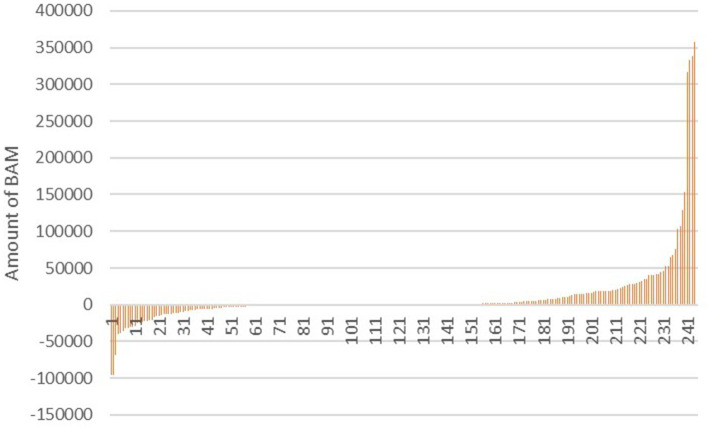
Reported annual profit of the veterinary practices in Bosnia and Herzegovina in the year 2021.

Out of the total veterinary practices surveyed (*n* = 244), a subset of 50 practices (20.49%) was identified with at least one blocked financial account, leading to a lack of reported revenue during the study period. 72 (29.51%) reported a negative annual profit, indicating financial challenges within the industry. On the other hand, 122 practices (50%) reported a profitable business, suggesting a significant portion of veterinary practices in the region were able to sustain profitability (Figure 5).

**Figure 6:**
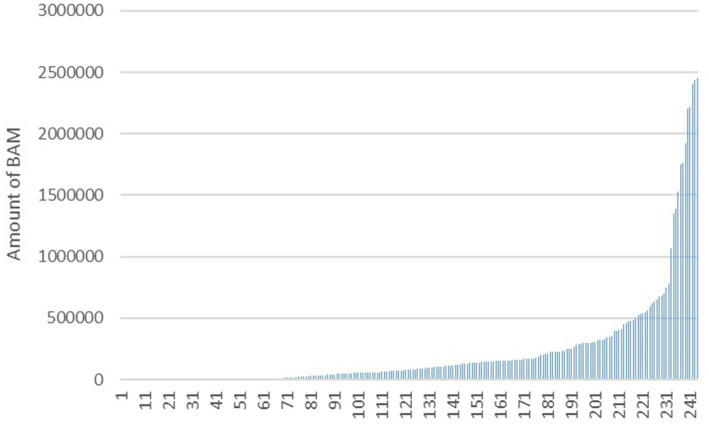
Reported annual revenue of the veterinary practices in Bosnia and Herzegovina in the year 2021.

The mean annual revenue across the surveyed practices was found to be 278,641 BAM, while the mean annual profit was 12,123 BAM. These figures provide valuable insights into the financial health of veterinary practices in Bosnia and Herzegovina during the study period. A closer examination of the annual revenue distribution revealed that a substantial majority of businesses (86.6%) reported revenues of less than 500,000 BAM. This suggests that a significant proportion of veterinary practices in the region operated on relatively modest revenue streams, with only a minority generating higher annual incomes (Figure 6).

Regarding employment characteristics, the majority of veterinary practices (53.1%) had fewer than four employees, reflecting the prevalence of small-scale practices in the region. Conversely, a smaller fraction (9.3%) of practices had a more extensive workforce of 10 or more employees. The average number of employees per veterinary practice in Bosnia and Herzegovina was 5.29, indicating that, on average, these practices operated with a moderately sized workforce.

To explore the influence of digital factors on the financial performance of veterinary practices, a statistical analysis was conducted. The results indicated that website presence, SEO optimization, social media presence, continuous administration of social media, website Google advertisement, and social media advertisement had a positive impact on the annual profit of businesses. Further analysis was conducted to assess the effect sizes of these digital factors on the annual profit. Based on the Hedges’ *g* test, large effect sizes were observed with SEO optimization, Google advertisement, and social media advertisement, suggesting that these factors significantly contributed to increased annual profits. On the other hand, website presence, social media presence, and continuous administration of social media showed smaller effect sizes, indicating a relatively less pronounced but still noteworthy impact on annual profits (Table 2). Businesses with a strong digital presence, effective SEO implementation, and well-managed social media accounts appear to experience better financial outcomes. Moreover, engaging in online advertising through Google and social media platforms has the potential to significantly boost profitability.

**Table 2:** Statistical analysis of the effects of investigated independent variables on the reported annual profit (dependent variable) of veterinary businesses.

Independent variables	Annual profit (dependent variable) stratified by categories of independent variables						
	*n*	Mean	SD	*t*	*df*	*p*	Hedges’ *g*
Have website	25	35870.24	88125.07	2.3556	192	0.0195^*^	0.504
No website	169	8609.87	47158.04				
Social media present	134	17281.55	63237.70	2.3409	192	0.0203^*^	0.363
Social media absent	60	−2207.30	18245.99				
Continuously administered social media	92	28322.37	71481.24	3.0855	132	0.0025^*^	0.575
Not continuously administered social media	42	−6903.1	27548.94				
Professionally made website	19	47262.95	98681.46	1.1584	23	0.259	0.542
Non-professionally made website	6	−206.67	12843.38				
Social media advertisement	19	74644.84	114681.2	4.5771	132	<0.0001^*^	1.13
No social media advertisement	115	7804.14	44157.4				
Search engine optimized websites	7	108,474	148263.9	2.9534	23	0.0071^*^	1.32
Not search engine optimized websites	18	7635.44	13811.05				
No website—continuously administered social media	77	22812.3	60906.37	2.9802	109	0.0036^*^	0.614
No website—not continuously administered social media	34	−9909.76	29127.52				
Website Google advertisement	4	178442.3	169469.2	4.9849	23	<0.0001^*^	2.72
No website Google advertisement	21	8713.67	13097.74				
Existent Google business account	117	18325.66	66731.10	1.9635	192	0.051	0.288
Non-existent Google business account	77	2697.69	25206.09				
Google business rating =5	87	20002.74	75259.37	1.8222	192	0.07	0.263
Google business rating ≤4	107	5715.76	27349.00				

^*^—Statistical significance; blue—*p* < 0.05 and Hedges’ *g* >0.8; green—*p* < 0.05 and Hedges’ *g* ≤ 0.8; white—*p* ≥ 0.05.

## 4. Discussion

Urbanization and growing pet ownership (2) had led to a shift in the focus of the profession and significant development of veterinary services offered to satisfy the needs of the owners of the growing population of companion animals (6). Leighton (7) was already alarmed that the veterinary profession in North America has become severely imbalanced, changing the focus to companion animal practice and it seems this became a global reality. This market-driven approach has a severe impact on the veterinary profession leaving society as a whole poorly served, with fundamental social issues, such as public health, food safety, food supply, and environmental security, unaddressed by the animal health specialist society has educated at great expense. This opinion was supported more recently by other authors as well (3, 8).

Digital technology, including novel telehealth, web-based tools, and artificial intelligence, is becoming increasingly important in the veterinary profession as a way to improve its efficiency and offer better services more easily accessible and available for all our stakeholders (9, 10). A review of existing, but still rare, literature, along with our study, supported our hypothesis that the effective and fast adoption of information technologies in veterinary services is correlated to the improved business performance of veterinary practices.

The present study unveiled that a majority (89.8%) of veterinary practices lack a website associated with their business, with a significant disparity in annual profit between veterinary practices with and without a website. Furthermore, SEO had large effect on annual profit amongst the website-owning veterinary practices in Bosnia and Herzegovina, being one of the most influential variables on the measured outcome (Table 2). These findings are consistent with previous studies that have examined the impacts of websites and SEO on business outcomes, as reported in studies conducted by Tomasi and Li (11) and Jones et al. (12) which focused on small to medium size enterprises (SMEs) from various niches, indicating that veterinary practices without a website, or without an SEO optimized website, may experience similar effects as businesses in other industries. Notably, as revealed in this study, the professional development status of the website has had no measurable impact on the annual profit of veterinary practices in Bosnia and Herzegovina.

Social media presence was found to be more widespread among veterinary practices, with 54.9% of practices having a presence on at least one platform, and Facebook being the most popular choice. Social media provides businesses with the opportunity to engage, learn from, and listen to their potential customers, as noted in previous studies (12). In the current study, the impact of social media on the annual profit of veterinary practices in Bosnia and Herzegovina was found to be significant, but providing a medium-sized effect on the dependent variable (Table 2). None of the practices had deployed a mobile application on either the Apple App Store or Google Play Store. This lack of mobile app adoption indicates a missed opportunity to engage with tech-savvy clients and provide them with convenient access to veterinary services through mobile devices.

In Bosnia and Herzegovina, businesses often face challenges and are comparatively behind in terms of business development to their equivalents in other European countries. This phenomenon is most likely influenced by a wide range of factors including culture, history, and politics (13). This trend is also reflected in the field of online marketing, where only a small proportion, specifically 8.2% (*n* = 20), of veterinary practices conducted online marketing campaigns, in contrast to a much higher percentage of 91% and 56% observed among SMEs in the United States (14) and European Union (15), respectively.

The majority of businesses in the present study could be categorized as SMEs by a number of employees, with less than 4 people employed in over 50% of the practices surveyed. The mean (519,224) and median (123,209) of the annual revenue is relatively high for small businesses as veterinary practices are (i.e., considering the number of employees), suggesting that a few extreme outlines in overall veterinary businesses are driving up the average. This confirms the standard deviation of both the annual profit and annual revenue, indicating that there is a lot of variability in the data. The findings of the present study highlight the financial difficulties faced by veterinary practices in Bosnia and Herzegovina in 2021 with half (*n* = 122) reporting either no revenue or negative annual profit. However, these findings are susceptible to further evaluation, since the tax burden is heavy for the SMEs in Bosnia and Herzegovina (16). Businesses in Bosnia and Herzegovina often resort to strategies such as partial revenue disclosure or implementation of envelope wages, as noted by Kurta et al. (17). While there is a lack of studies specifically examining these practices in the veterinary industry, it is not implausible to consider the possibility of similar scenarios within this particular business niche.

The study confirmed that a positive impact on annual profit is correlated with the implementation of effective digital marketing strategies, including website presence, SEO, Google business account existence, website Google advertisement, social media presence, continuous administration of social media, and social media advertisement. Building online presence is becoming one of the most important things that veterinary services can do to promote themselves with simple steps such as creating quality content and maintaining consistently good service feedback (18). However, our analysis indicates that SEO, Google advertisement, and social media advertisement had a large effect on annual profit, while website presence, social media presence, and continuous administration of social media had a medium size effect on annual profit.

The use of web and web-based tools including telehealth as part of veterinary services has been a growing topic of interest, particularly with the advancement of digital information and communication technologies (9, 19). Web and social media presence are not only part of marketing but it might also be considered as a way to offer better or improved services (advice, communication, education, building the trust between clients and veterinarians). It saves time and optimizes resources and might enable a better balance of the veterinary workforce. Clarke and Chapman (20) have emphasized that marketing involves not only advertising and special offers but also the clients, customers, and staff of veterinary practices. A balanced customer relationship must be combined with the knowledge of staff and veterinarians. While corporate companies recognize the significance of marketing and employ marketing specialists to analyze market trends and develop new services and products to boost sales, veterinary practices generally do not have such specialists or actively engage them. However, employing marketing specialists in veterinary practices in Bosnia and Herzegovina may not be advantageous due to the majority of businesses being SMEs and consequently not having sufficient funds. It would be beneficial if professional veterinary associations in Bosnia and Herzegovina actively promoted online marketing tools, offered introductory support for business startups, and provided guidelines on creation of successful business and marketing strategies for veterinary practices looking to venture into the online marketing sector. Nonetheless, it is crucial to understand the needs of customers and adapt to their changing requirements, as this is the key to the success of veterinary practice marketing (21).

## 5. Conclusion

The findings of our study provide evidence that digital presence and information technology adoption have a significant impact on the business performance of veterinary practices in Bosnia and Herzegovina. The low percentage of veterinary practices with a website (10.2%) and the limited use of digital marketing strategies highlight the missed opportunities for practices to leverage digital technologies to enhance their business performance. The data analysis demonstrates a positive association and size of effect between effective digital marketing strategies, such as website presence, SEO, and social media advertisement, and increased annual profit among veterinary practices.

These findings may serve as valuable insights for veterinary practitioners, professional associations, and policymakers in Bosnia and Herzegovina and other countries with similar socio-economic contexts to develop guidelines and support mechanisms for the effective adoption and implementation of digital technologies in veterinary practices. However, conducting an in-depth analysis of the wider implementation of information technologies and their potential impact on enhancing business performance, as well as meeting the evolving needs of clients and society, is needed. Further studies might address whether and how digital technologies could serve as a solution to overcome the existing shortage of veterinary workers and the imbalance in providing adequate coverage of the existing and future needs of both clients and society. However, our results highlight and support the urgent need to elaborate effective strategies for integrating novel knowledge and skills related to digital technologies into the day-one competencies of veterinary graduates.

## Data availability statement

The raw data supporting the conclusions of this article will be made available by the authors, without undue reservation.

## Ethics statement

The following Ethics Statement serves to confirm that the research conducted in this study did not involve any experiments on animals. Additionally, all data utilized in this research is publicly available and can be accessed by anyone who wishes to verify or replicate the findings. Therefore, ethical approval was not required for this study.

## Author contributions

NF and AM: study design. AM, NF, EM, and SŠ-H: data collection, formal analysis, and writing—original draft. AM, NF, and EM: data curation. All authors contributed to the article and approved the submitted version.

## Conflict of interest

The authors declare that the research was conducted in the absence of any commercial or financial relationships that could be construed as a potential conflict of interest.

## Publisher’s note

All claims expressed in this article are solely those of the authors and do not necessarily represent those of their affiliated organizations, or those of the publisher, the editors and the reviewers. Any product that may be evaluated in this article, or claim that may be made by its manufacturer, is not guaranteed or endorsed by the publisher.
